# The Role of Immunonutrition in Patients

**DOI:** 10.3390/nu15030780

**Published:** 2023-02-03

**Authors:** Marco Cintoni, Maria Cristina Mele

**Affiliations:** 1 UOC di Nutrizione Clinica, Dipartimento di Scienze Mediche e Chirurgiche, Fondazione Policlinico Universitario A. Gemelli IRCCS, Largo A. Gemelli 8, 00168 Rome, Italy; 2 Dipartimento di Medicina e Chirurgia Traslazionale, Università Cattolica del Sacro Cuore, 00168 Rome, Italy

Immunonutrition (IN) is defined as “the use of specific nutritional substrates, called «immunonutrients» having the ability of modulating specific mechanisms involved in several immune and inflammatory pathways” [1]. The most studied immunonutrients are ω-3 fatty acids, glutamine, sulfur-containing amino acids, antioxidants, arginine, and nucleotides alone or in various combinations [2].

Their potential role in elective surgery has been well documented: the benefits have also been supported by several very recent systematic reviews and meta-analyses, revealing a reduced risk of complications, lower length of hospital stays, and reduced mortality [3,4,5]. IN has shown its efficacy also in improving clinical outcomes in acute pancreatitis [6], critical illness [7], acute respiratory distress syndrome [8], and sepsis [9].

Moreover, recent studies analyzed the impact of oral IN on the changes in the tumor microenvironment. In particular, in a prospective study, D’Ignazio et al. enrolled 24 patients affected by gastrointestinal neoplasms, which showed higher T-helper and Cytotoxic lymphocytes, a lower number of exhausted and regulatory phenotype lymphocytes, M1 polarization with a lower number of CD163+ macrophages, and inhibition of the PD-1/PD-L1 pathway [10]. The same conclusion was also obtained by Molfino et al., who enrolled 12 patients with gastric cancer, observing a tendency to increase the number of T-lymphocytes CD8+, CD83+, CD68+, an absence of F4/80+ cells, a positive correlation between CD8+ and macrophages CD68+, and a significant correlation between CD68+ and CD40+, with a global change in inflammatory patterns [11]. Moreover, recent literature supports the hypothesis that IN supplementation can be effective in patients undergoing chemoradiotherapy, reducing the rate of adverse reactions, especially mucositis [12]. At the same time, the role of IN on oncological patients undergoing immunotherapies seems to be an interesting field of research [12].

On the other hand, IN impact on premature neonates [13], pediatric food allergy [14], atherosclerosis [15], and the elderly [16] is still under debate, and more studies are needed to clarify its effect on those populations.

Papers that cover all the challenges and possible influences that IN can potentially have on several clinical conditions are welcomed to the current Special Issue entitled “The Role of Immunonutrition in Patients”, which aims to analyze and develop solutions and support patients in different clinical settings.

## References

[1] Gianotti L., Nespoli L., Sandini M. (2022). Pharmaconutrition: Which substrates?. Eur. J. Surg. Oncol..

[2] Yu K., Zheng X., Wang G., Liu M., Li Y., Yu P., Yang M., Guo N., Ma X., Bu Y. (2020). Immunonutrition vs Standard Nutrition for Cancer Patients: A Systematic Review and Meta-Analysis (Part 1). J. Parenter. Enter. Nutr..

[3] Wang S.-Y., Hung Y.-L., Hsu C.-C., Hu C.-H., Huang R.-Y., Sung C.-M., Li Y.-R., Kou H.-W., Chen M.-Y., Chang S.-C. (2021). Optimal Perioperative Nutrition Therapy for Patients Undergoing Pancreaticoduodenectomy: A Systematic Review with a Component Network Meta-Analysis. Nutrients.

[4] Slim K., Badon F., Vacheron C.H., Occean B.V., Dziri C., Chambrier C. (2022). Umbrella review of the efficacy of perioperative immunonutrition in visceral surgery. Clin. Nutr. ESPEN.

[5] Niu J.W., Zhou L., Liu Z.Z., Pei D.P., Fan W.Q., Ning W. (2021). A Systematic Review and Meta-Analysis of the Effects of Perioperative Immunonutrition in Gastrointestinal Cancer Patients. Nutr. Cancer.

[6] Jafari T., Feizi A., Askari G., Fallah A.A. (2015). Parenteral immunonutrition in patients with acute pancreatitis: A systematic review and meta-analysis. Clin. Nutr..

[7] Marik P.E., Zaloga G.P. (2008). Immunonutrition in critically ill patients: A systematic review and analysis of the literature. Intensiv. Care Med..

[8] Dushianthan A., Cusack R., Burgess V.A., Grocott M.P., Calder P. (2019). Immunonutrition for acute respiratory distress syndrome (ARDS) in adults. Cochrane Database Syst. Rev..

[9] Wang H., Su S., Wang C., Hu J., Dan W., Peng X. (2022). Effects of fish oil-containing nutrition supplementation in adult sepsis patients: A systematic review and meta-analysis. Burn. Trauma.

[10] D’Ignazio A., Kabata P., Ambrosio M.R., Polom K., Marano L., Spagnoli L., Ongaro A., Pieretti L., Marrelli D., Biviano I. (2020). Preoperative oral immunonutrition in gastrointestinal surgical patients: How the tumour microenvironment can be modified. Clin. Nutr. ESPEN.

[11] Molfino A., Mari A., Paldino A., Carletti R., Imbimbo G., Cardi M., di Gioia C.R.T., Laviano A. (2023). Effects of oral immunonutrition on histological changes of inflammatory infiltration of the tumor microenvironment among patients with a new diagnosis of gastric cancer. Nutrition.

[12] Lyra M.M.F., Meira J.E.C., Guedes G.D.S., Bueno N.B. (2021). Immunonutrition in head and neck cancer: Systematic review and metanalysis of its clinical and nutritional effects. Clin. Nutr. ESPEN.

[13] Pilotto S., Agustoni F., Morelli A.M., Lobascio F., Cereda E., Bironzo P., Trestini I., Milella M., Novello S., Pedrazzoli P. (2022). Nutritional support in lung cancer: Time to combine immunonutrition with immunotherapy?. Nutrition.

[14] Walsh V., McGuire W. (2019). Immunonutrition for Preterm Infants. Neonatology.

[15] Coppola S., Carucci L., De Michele R., Berni Canani R. (2022). The potential role of preventive and therapeutic immunonutrition strategies for pediatric food allergy: A mini-review. Front. Nutr..

[16] Gonçalves T.J.M., Gonçalves S.E.A.B., Nava N., Jorge V.C., Okawa A.M., Rocha V.A., Forato L.C.H., Furuya V.A.O., Martins S.S., Oksman D. (2021). Perioperative Immunonutrition in Elderly Patients Undergoing Total Hip and Knee Arthroplasty: Impact on Postoperative Outcomes. J. Parenter. Enter. Nutr..

